# Establishment of an extracorporeal cardio-pulmonary resuscitation program in Berlin – outcomes of 254 patients with refractory circulatory arrest

**DOI:** 10.1186/s13049-020-00787-w

**Published:** 2020-09-23

**Authors:** Jens Nee, Roland Koerner, Daniel Zickler, Tim Schroeder, Philipp Enghard, Lutz Nibbe, Dietrich Hasper, Robert Buder, Christoph Leithner, Christoph J. Ploner, Kai-Uwe Eckardt, Christian Storm, Jan M. Kruse

**Affiliations:** 1 Department of Nephrology and Medical Intensive Care, Charité-Universitätsmedizin Berlin, Augustenburger Platz 1, 13353 Berlin, Germany; 2 Department of Emergency and Intensive Care Medicine, Ernst von Bergmann Klinikum, Charlottenstraße 72, 14467 Potsdam, Germany; 3 Department of Neurology and Experimental Neurology, Charité-Universitätsmedizin Berlin, Augustenburger Platz 1, 13353 Berlin, Germany

**Keywords:** eCPR, Refractory cardiac arrest, Extracorporeal life support

## Abstract

**Objective:**

Optimal management of out of hospital circulatory arrest (OHCA) remains challenging, in particular in patients who do not develop rapid return of spontaneous circulation (ROSC). Extracorporeal cardiopulmonary resuscitation (eCPR) can be a life-saving bridging procedure. However its requirements and feasibility of implementation in patients with OHCA, appropriate inclusion criteria and achievable outcomes remain poorly defined.

**Design:**

Prospective cohort study.

**Setting:**

Tertiary referral university hospital center.

**Patients:**

Here we report on characteristics, course and outcomes on the first consecutive 254 patients admitted between August 2014 and December 2017.

**Intervention:**

eCPR program for OHCA.

**Mesurements and main results:**

A structured clinical pathway was designed and implemented as 24/7 eCPR service at the Charité in Berlin. In total, 254 patients were transferred with ongoing CPR, including automated chest compression, of which 30 showed or developed ROSC after admission. Following hospital admission predefined in- and exclusion criteria for eCPR were checked; in the remaining 224, 126 were considered as eligible for eCPR.

State of the art postresuscitation therapy was applied and prognostication of neurological outcome was performed according to a standardized protocol.

Eighteen patients survived, with a good neurological outcome (cerebral performance category (CPC) 1 or 2) in 15 patients. Compared to non-survivors survivors had significantly shorter time between collaps and start of eCPR (58 min (IQR 12–85) vs. 90 min (IQR 74–114), *p* = 0.01), lower lactate levels on admission (95 mg/dL (IQR 44–130) vs. 143 mg/dL (IQR 111–178), *p* <  0.05), and less severe acidosis on admission (pH 7.2 (IQR 7.15–7.4) vs. 7.0 (IQR6.9–7.2), *p* <  0.05). Binary logistic regression analysis identified latency to eCPR and low pH as independent predictors for mortality.

**Conclusion:**

An eCPR program can be life-saving for a subset of individuals with refractory circulatory arrest, with time to initiation of eCPR being a main determinant of survival.

## Introduction

The prognosis of out of hospital circulatory arrest (OHCA) remains poor even in countries with well established emergency medical service (EMS). However, a certain proportion of patients is refractory to advanced cardiac life support (ACLS) and the chances for a return of circulation (ROSC) decline with resuscitation time [1]. In such inevitably fatal cases extracorporeal CPR (eCPR) using veno-arterial exracorporeal membrane oxygenation (ECMO) can provide life-saving bridging and enable identification of the course of circulatory arrest and causal therapy. Current guidelines, which fail to clarify ideal inclusion and exclusion criteria, recommend the invasive and aggressive approach of eCPR for very specific cases, and emphasize the necessity of a highly trained team [2].

In this setting we have implemented an eCPR pathway at the Circulatory Arrest Center (CAC) of the Charité-Universitätsmedizin Berlin (Campus Virchow Klinikum) in 2014. After intensive skill training of the staff and establishment of standardized procedures, the EMS was offered the opportunity to transfer patients without ROSC during ongoing CPR to the center for potential rescue eCPR.

The aim of the current analysis is to describe our experience and patient outcomes and to identify characteristics associated with good prognosis in patients started on eCPR.

## Materials and method

We performed a retrospective analysis of the routine clinical data from 254 patients admitted to our center during ongoing CPR for eCPR between August 2014 and December 2017. The local ethics committee has approved this analysis.

### Intensive Care Unit (ICU) setting

The eCPR programm was established at an interdisciplinary medical intensive care unit (ICU) with a patient capacity of 24. After establishment of a CAC, an eCPR (veno-arterial ECMO) program was implemented. A 24/7 telephone hotline was established for the EMS to directly reach the intensivist on duty in the CAC and to announce transfer of a patient under CPR. A short checklist was used to obtain important information from the EMS (time of ongoing CPR, location, use of a mechanical chest compression device for transport, initial rhythm, patient age (frailty), prior history, bystander CPR) during the notification call. The final decision whether or not to transport the patient under ongoing mechanical chest compression was left to the EMS team on scene. Within a preparation time of 5–10 min the eCPR team was ready to accept the patient on the ICU.

### eCPR team

**Fig. 1:**
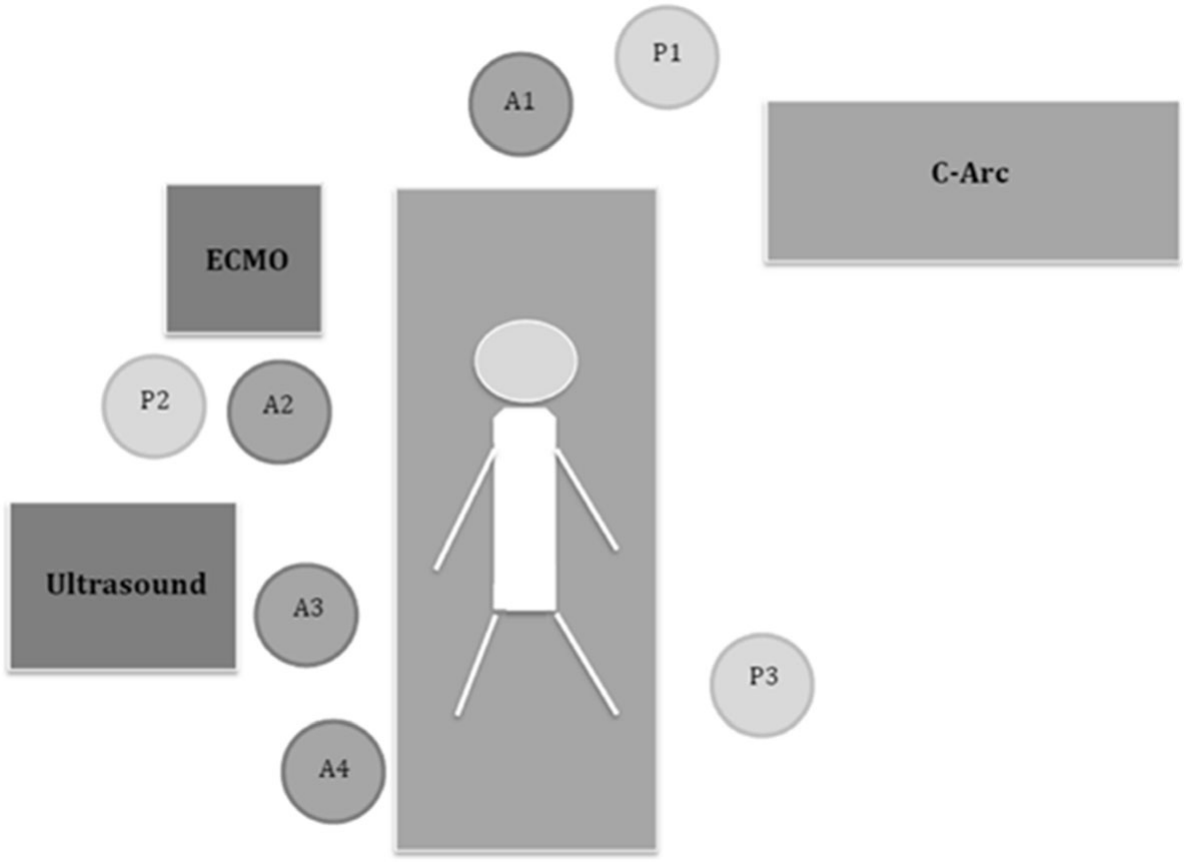
eCPR - setting and team composition: (A1) consultant in ICU training, leader of CPR; (A2) attending physician, pump operator, supervisor; (A3) intensivist, responsible for canulation; (A4) physician in ICU training; (P1-P3) nurses specialized in intensive care; ECMO extracorporeal membrane oxygenation, C-Arc mobile X-ray unit

The team was availabale 24/7 and comprised at least one consultant (leader of CPR team), an attending physician (pump operator, supervisor), an intensivist (responsible for cannulation) and a physician in ICU training. In addition three nurses specialized in intensive care were part of the team. We implemented local standard operating procedures with detailed workflow for the team during eCPR. The team had extensive skill training with a full-scale simulator (manikin) for simulation of admission, CPR, ultrasound-guided vessel cannulation, and priming and start of the eCPR pump. Standard operating procedures also defined the position of each team member in the intervention room and his or her personal tasks and responsibility at any time (Fig. 1).

### eCPR procedure

Upon EMS arrival, one team (physician in ICU training and nurse) was responsible for patient ventilation, administration of epinephrine and other drugs, monitoring, and defibrillation if indicated, all according to current ACLS guidelines. A focused clinical examination and transthoracic echocardiography was performed.

Taking the first blood gas analysis, the whole team decided together during a short time-out whether to start eCPR or to cease CPR according to the exclusion criteria for eCPR. Patients were excluded from eCPR if cardiac arrest was not witnessed, no bystander CPR was performed, the patient had active malignant disease or was frailty, the presumed time to eCPR exceeded 90 min, the EMS team used no mechanical compression device for transport, a technical impossibility for cannulation (diameter of the vessels) or existence of critically bleeding (e.g. esophageal bleeding, see Table 1).

**Table 1:** Checklist - Inclusion Criteria for eCPR in our Center

*EMS:*	
Cardiac arrest is witnessed	
Bystander CPR	
No active malignant disease	
No frailty	
The presumed time to eCPR less than 90 min	
CAVE: Use of mechanical compression device (mandatory for transport)	
*Additionally after admission in hospital:*	
Technical possibility for cannulation (diameter of the vessels)	
No critically bleeding	

At the time of eCPR decision, the coronary angiography team was informed. The ICU specialist and the other two nurses were responsible for obtaining vascular access to the femoral vessels under ultrasound guidance. Cannulation of the femoral vein and artery was performed with 15–17 French (artery) and 23 French (vein) catheters, respectively. Following cannulation eCPR settings comprised a minimum blood flow of 3 L/min and a minimum gas flow of 3 L/min. After connection of the device to the patient, the automated CPR device was removed and eCPR device settings were adjusted. Via ultrasound guidance, a standard arterial blood pressure catheter was placed into the right radial artery to measure pressure generated by the laminar pump flow and to calculate oxygen extraction.

To reduce the risk of catheter-associated infections after cannulation, a standardized antibiotic regimen (carbapenem and glycopetide) was initiated in all patients.

### Diagnostic procedures

After ECLS initiation, patients with a suspected cardiac cause of arrest underwent immediate coronary angiography and, if needed, rescue percutaneous coronary intervention (PCI).

If a myocardial infarction was ruled out, a CT scan was initiated for further examination.

In approximately half the cases a brain CT and trauma sequence were performed as well to rule out intracranial hemorrhage (ICH) or severe brain edema.

### Postresuscitation care

Postresuscitation management was conducted, including targeted temperature management at 33 °C for at least 24 h followed by a slow warming with 0.25 °C/h in all patients. Cooling was started as soon as possible after ECMO-implantation regardless wether patients developed ROSC or not. Blood temperature was influenced within the external circuit. A bladder pressure probe was installed to monitor for abdominal compartment syndrome. Patients regaining ROSC were weaned from the eCPR pump according to our local protocol.

### Prognostication of neurological outcome

Neurological prognostication followed a local standard protocol, in line with current guidelines for prognostication in post cardiac-arrest patients [3]. In addition we made a routine CT scan as a part of the neurological outcome assessment such as determination of the grey – white matter ratio or other signs of hypoxic brain damage 72 h after arrest at the earliest. Outcome was assessed at discharge by using the Pittsburgh Cerebral Performance Category (CPC). In the subgroup of patients being discharged, subsequent mortality was assessed using the online registry of Berlin citizens.

### Withdrawal of treatment

In patients with severe brain edema and evidence for poor neurological outcome eCPR was withdrawn or subsequent intensive management was ceased after discussion with the family.

### Statistics

Results are given depending on their scale in proportions (%), median with 25–75% quartiles (interquartile range [IQR]), or arithmetic mean with 95% confidence interval (95% CI). Statistical significance was tested using two-tailed Student t test, Wilcoxon-Mann-Whitney test, or Fisher exact test, as appropriate. A *p*-value of less than 0.05 was considered statistically significant. Binary logistic regression analysis was used to identify independent predictors for mortality. Kaplan-Meier analysis was used for the 12-month follow-up. All analyses were performed with SPSS (IBM® SPSS® Statistics version 25).

## Results

**Fig. 2:**
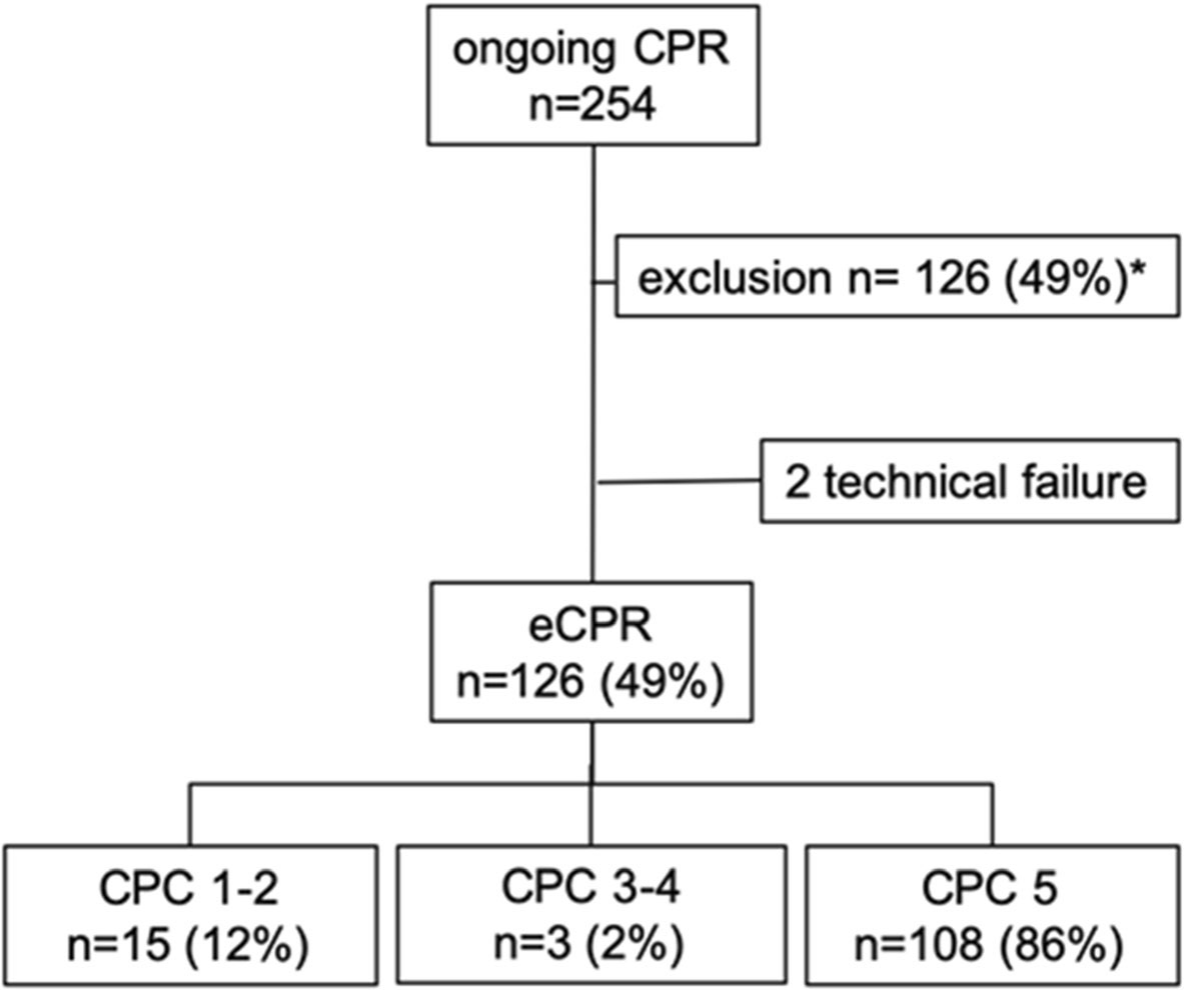
Flowchart of cardiac arrest patients. The transfer under CPR was made by using an automated CPR devise (LUCAS® or Autopuls®). *30 spontaneous ROSC during admission. Ninety-six not fullfilling inclusion criteria for eCPR. ROSC return of spontaneous circulation, OHCA out-of-hospital cardiac arrest, CPR cardiopulmonary resuscitation, ECLS veno-arterial extra corporal membrane oxygenation, CPC cerebral performance category. VF ventricular fibrillation, PEA pulseless electrical activity, CA cardiac arrest, ACS acute coronary syndrome

Between August 2014 and December 2017, a total of 254 patients were admitted for eCPR mainly via EMS under ongoing mechanical CPR. Most patients suffered from out of hospital caridac arrest, a minority of 50 patients had refractory in house hospital cardiac arrest and were admitted on ICU via the hospital resuscitation team. Of those 30 patients showed or developed ROSC during the admission procedure. In 128 of the remaining 224 patients, the decision was made to initiate eCPR, whereas treatment was withdrawn in 126 patients. In two patients eCPR could not be initiated due to technical reasons (Fig. 2).

### Patient characteristics of admitted patients

Table 2 shows detailed characteristics of the 224 patients admitted under ongoing resuscitation who had not developed ROSC, stratified according to whether a decision was taken to attempt eCPR (128 patients) or resuscitation was discontinued (96 patients).

**Table 2:** Detailed characteristics of included and excluded patients for eCPR intervention

Characteristics of included and excluded patients				
Variable	All ***n*** = 224	Inclusion ***n*** = 128	Exclusion ***n*** = 96	***p***-value
Gender (male)	175 (78.1%)	94 (73.4%)	81 (84.4%)	n.s.
Age (years)	54 (43–64)	52 (42–60.75)	56 (46–69)	< 0,05
OHCA	174 (77.7%)	87 (68%)	87 (90.6%)	< 0,05
EMS first rhythm (shockable)	6 (42.86%)	64 (50%)	32 (33.3%)	n.s.
Rhythm on admission (shockable)	52 (23.2%)	33 (25.78%	19 (19.79%)	n.s.
Epinephrine (mg; total amount)	7 (5–10)	7 (4–10)	8 (5–10)	n.s.
APACHE (admission)	42 (38–46)	41 (38–45.75)	43 (38–47)	n.s.
Collapse to admission (min)	63.5 (50–80)	57 (45–67)	75 (60–90)	< 0,05
Admission laboratory values				
pH	6.93 (6.8–7.14)	7.06 (6.89–7.2)	6.8 (6.7–6.97)	< 0,05
Lactate	143 (111.5–185)	137 (105–172)	153.5 (122.25–196.75)	< 0,05
Potassium	4.6 (3.9–5.8)	4.3 (3.75–5.1)	5.1 (4.23–6.6)	< 0,05
INR	1.79 (1.49–3.07)	1.81 (1.49–3.22)	1.79 (1.41–2.74)	n.s.

Data are given as median (25–75% interquartile range) or absolute numbers

*OHCA* out-of-hospital cardiac arrest, *EMS* emergency medical service, *APACHE* Acute Physiology And Chronic Health Evaluation, *INR* international normalized ratio

### Baseline characteristics of eCPR patients

Table 3 shows the baseline characteristics and treatment parameters of the 126 patients in whom eCPR was initiated stratified by survival to discharge status. Gender and age were equally distributed between survivors and non-survivors; the majority of patients had an OHCA with a shockable first rhythm (Table 3). Detailed analysis concerning etiology of cardiac arrest depending on initial rhythm in survivors is given as supplemental table (Table S 1).

**Table 3:** Baseline characteristics and treatment parameters of patients in whom eCPR was initiated stratified by survivor status

Baseline characteristics				
Variable	All ***n*** = 126	Non-survivors ***n*** = 108	Survivors ***n*** = 18	***p***-value
Gender (male)	93 (74%)	79 (73%)	14 (78%)	n.s.
Age (years)	52 (42–61)	52 (41–59)	59 (46–66)	n.s.
OHCA	85 (67,5%)	77 (71%)	8 (44%)	< 0,05
EMS first rhythm (shockable)	64 (51%)	53 (49%)	11 (61%)	n.s.
Rhythm on admission (shockable)	32 (25%)	24 (22%)	8 (44%)	< 0,05
Epinephrine (mg; total amount)	7 (4–10)	7 (4–11)	5 (1–7)	< 0,05
Suspected cause of arrest				
Cardiac	76 (60%)	64 (59%)	12 (67%)	n.s.
Non-cardiac	50 (40%)	44 (41%)	6 (33%)	
APACHE (admission)	41 (38–46)	42 (38–47)	39 (37–43)	n.s.
Ventilator time (hours)	25 (6–86)	15 (6–59)	722 (242–1003)	< 0.05
eCPR related time intervals				
Collapse to admission (min)	57 (45–67)	60 (46–70)	33 (13–53)	< 0,05
Collapse to eCPR (min)	89 (73–111)	90 (74–114)	58 (12–85)	< 0,05
ECPR duration (hours)	20.7 (5.1–63)	12 (5–44.1)	89 (49–153.7)	< 0.05
Admission laboratory values				
pH	7.1 (6.9–7,2)	7 (6.9–7.2)	7.2 (7.15–7.4)	< 0.05
Lactate	137 (104–173)	143 (111–178)	95 (44–130)	< 0,05
Potassium	4.3 (3.7–5.1)	4.4 (3.8–5.2)	4 (3.5–4.5)	< 0,05
INR	1.8 (1.5–3.2)	2 (1.5–3.7)	1.5 (1.3–2.4)	< 0,05
Blood and volume treatment during the 1st 24 h				
Red cell units	4 (2–10)	5.5 (2–12)	2.5 (0–4.5)	< 0,05
FFP (units)	5 (0–12)	6 (0–12)	4 (0–10.3)	n.s.
Volume first 24 h (liter)	5 (2.1–8)	5.3 (2.3–8.2)	2.9 (2.1–5.9)	n.s.
Diagnostic procedures				
Coronary angiography	82 (65%)	69 (64%)	13 (72%)	n.s.
Relevant coronary stenosis	67 (53%)	56 (52%)	11 (61%)	n.s.
CT (full body scan)	63 (50%)	49 (45%)	14 (78%)	< 0,05
Normal result	23 (18%)	13 (12%)	10 (56%)	
ICH	5 (4%)	4 (4%)	1 (6%)	
Brain edema	21 (17%)	20 (19%)	1 (6%)	
Pulmonary embolism	7 (6%)	6 (6%)	1 (6%)	
Aortic dissection	3 (2%)	3 (3%)	0	

Data are given as median (25–75% interquartile range) or absolute numbers

*OHCA* out-of-hospital cardiac arrest, *EMS* emergency medical service, *CA* cardiac arrest, *APACHE* Acute Physiology And Chronic Health Evaluation, *eCPR* extracorporeal cardiopulmonary resuscitation, *INR* international normalized ratio, *EK* erythrocyte concentrate, *FFP* fresh frozen plasma, *CT* computer tomography, *ICH* intracranial hemorrhage

### Time intervals in eCPR patients

Survivors had a shorter time from beginning of collapse to admission (33 min (IQR 13–53) vs. 60 min (IQR 46–70), *p* <  0.05) as well as a shorter time from beginning of collapse to the start of eCPR (58 min (IQR 12–85) vs. 90 min (IQR 74–114), *p* <  0.05).

### Initial laboratory values in eCPR patients

Time-of-admission laboratory testing showed that survivors, compared to non-survivors, had lower lactate levels (95 mmol/L (IQR 44–130) vs. 143 mmol/L (IQR 111–178), *p* <  0.05) and higher pH levels (7.2 (IQR 7.15–7.4) vs. 7.0 (IQR6.9–7.2), *p* <  0.05).

### Treatment characteristics in eCPR patients

The need for blood volume resuscitation, erythrocyte transfusion, and fresh frozen plasma was lower in survivors compared to non-survivors (Table 3). Of all eCPR-qualifying patients, 82/126 received immediate coronary angiography (CAG) and 67/126 (53.2%) received PCI; there was no significant difference concerning frequency of CAG or PCI between survivors and non-survivors. An initial brain CT and trauma CT sequence was performed in 63/126 (50%). Intracranial hemorrhage (ICH) was found in 5 patients, severe brain edema in 21 patients, and aortic dissection in 3 patients.

Median duration of ECLS was 89 h (49–154) in survivors and 12 h (5–44) in non-survivors (*p* <  0.05). As expected, also the overall ICU treatment was longer in survivors, as reflected by a longer ventilation time.

### Outcome of eCPR patients

15/126 (11.9%) developed a good neurological outcome (CPC 1–2) and 3 patients (2.4%) were sent to neurorehabilitation with CPC 3–4. 108/126 (85.7%) patients died during eCPR due to development of a severe post-resuscitation syndrome including breakdown of the coagulation cascade and/or severe abdominal compartment syndrome (clinical presentation and intra-abdominal pressure > 20) and/or multi-organ failure. Decision to stop eCPR was taken in an interdisciplinary manner. Eighteen patients (14.3%) had no complications during the treatment, so that we could stop the eCPR therapy with development of a sufficient spontaneous circulation (monitored by echocardiography) and they survived ultimately.

Longer timing to eCPR (*p* <  0.05) and lower initial pH (*p* < 0.05) were identified as significant independent predictors for mortality in binary logistic regression analysis (Table 4).

**Table 4:** Binary logistic regression analysis with mortality as dependent variable

Model	B	Std. Error	Wald	Sig.t	Exp(B)
(Constant)	47.570	20.650			
Gender	0.696	1.189	0.343	0.558	2.006
Age	0.004	0.041	0.009	0.924	1.004
Cardiac cause	−0,143	1.155	0.015	0.902	0.867
Time to eCPR^a^	0.062	0.025	6.414	**0.011**	1.064
First pH	−6.340	2.869	4.883	**0.027**	0.002
First lactate	−0.012	0.011	1.125	0.289	0.988
Location^b^	−1.789	1.483	1.455	0.228	0.167

Dependent variable mortality; ^a^time from collapse to start of eCPR, ^b^ in-hospital circulatory arrest; *eCPR* extracorporeal cardiopulmonary resuscitation

### Follow-up of eCPR survivors

Follow-up was attempted at 12 months post-admission for all survivors (*n* = 18). Fourteen patients were alive, 2 patients had died within 12 months and 2 were lost to follow-up.

The Kaplan Meier analysis is given as supplemental figure (Fig. S 1).

### Patients regaining ROSC before / without eCPR

Of the 30 patients who showed ROSC during the admission procedure, 8 (27%) were discharged with a good outcome (CPC 1–2). One additional patient, who had been excluded from eCPR due to failure to fulfil inclusion criteria regained ROSC during admission procedures and experienced a good outcome (CPC 1).

## Discussion

This study reports the successful establishment of an eCPR program in a tertiary care setting. Our main findings with respect to patient outcomes are 1) survival and good neurological outcome is significantly related to timing of intervention, 2) prolonged latency from collapse to initiation of eCPR and lower pH at admission were independent factors predicting mortality in patients undergoing eCPR, 3) a relevant number of patients scheduled for eCPR regained ROSC after long-time CPR before or during hospital admission, and 4) patients surviving eCPR showed low mortality rates during a 12 month follow-up.

### Establishment of an eCPR program

We chose to systematically plan the establishment of an eCPR program including skill training of personnel involved and have built the program in the setting of a large tertiary care ICU. In our setting we have so far geared up the program to a frequency of approx. One to two admissions for eCPR per months. The overall rate of survival with good neurological outcome (CPC 1–2) achieved over a 4 year period among those in whom eCPR was initiated in our center was 12%.

Surprisingly a recent survey among USA ECMO centers in the Extracorporeal Life Support Organization (ELSO) registry revealed that almost all centers offering eCPR were academic (or teaching) hospitals but 60% perform less than 3 cases per year [4]. A retrospective analysis from the Vienna registry revealed that eCPR was performed in only seven patients out of 239 OHCA that fulfilled inclusion criteria, of whom only one patient had good neurological outcome [5]. On the other hand increasing numbers of centers report improved survival of OHCA patients treated with eCPR [6–8].

Obviously eCPR is not only associated with logistical but also substantial ethical challenges. Patient preferences are frequently unknown when CPR is started and information on medical history is limited. We have built our program on the opinion that the goal of eCPR should be to maximize chances for survival with good neurological outcome (CPC 1 or 2) while minimizing the chances for survival with poor neurological outcomes (CPC 3–5) or subsequent short-term mortality. Two strategies appear essential for achieving this goal: 1) careful and deliberate decisions to initiate (or not initiate) eCPR and 2) conducting subsequent ICU therapy (escalation, deescalation, withdrawl) based on neurological prognosis. In our views SOPs are helpful for both aspects.

### Inclusion criteria

Recommendations based on robust evidence concerning indication and inclusion criteria for eCPR are lacking. Threshold values for age, time interval since collaps and certain laboratory values are unavoidably arbitrary; systematic data collection, e.g. in international registries is urgently needed to establish a growing evidence base. In individual cases the inclination of teams to deviate from inclusion criteria and be less restrictive is understandable, in particular in the absence of strong evidence.

Surprisingly, many other eCPR centers do not seem to have formal inclusion and exclusion criteria and make case-by-case decisions regarding eCPR. As most studies used retrospective data with no formal inclusion and exclusion criteria, the interpretation of survival rates and outcome suffers a pre-selection bias that needs to be taken into account. Nevertheless a meta-analysis showed lower lactate, higher pH and shorter low-flow time to be associated with better outcome, in line with our results [9].

Bartos et al. reported their inclusion criteria for eCPR as follows:18 to 75 years of age, OHCA of presumed cardiac origin) initial rhythm of VF/VT, received 3 direct current shocks for VF/VT without ROSC or shock resulting in ongoing pulseless electrical activity or asystole, received amiodarone 300 mg, body habitus accommodating a Lund University Cardiac Arrest System automated CPR device, and estimated transfer time to the CCL of < 30 min [8].

In our center we regarded 90 min as the upper timelimit until patients had to be on ECMO wich translates into a maximum 60 min CPR until arrival at our ICU. Although the maximum age was higher with 75 years, they report a higher survival rate. This underlines the importance of CPR-duration for neurological prognosis of the patients and argues for a strict attachment to predefined inclusion criteria.

But even though we implemented a list of in- /exclusion criteria at our center as well for EMS-transport to the hospital with ongoing CPR as for ECMO-implantation after arrival in the hospital (Table 1), the final decision allways stays with the team in charge. Espeacially the EMS-Teams on scene did not always adhere to our recommendations and transported under ongoing CPR although inclusion criteria for ECMO-implantation were not met.

### Importance of time to inititation of eCPR

According to our data one of the main determinants of success is time between collapse and initiation of eCPR. This is consistent with reports in the literature. There is evidence that conventional CPR only achieves a maximum of 25% of normal cardiac output [10, 11]. Thus small retrospective studies and case series that revealed a survival benefit of eCPR compared to standard treatment demonstrated greater survival with shorter arrest-to-eCPR time interval [12, 13].

Wengenmaier et al. showed significant differences in survival between IHCA and OHCA patients; time to eCPR was significantly shorter in IHCA patients [14]. Other data comparing survival rates after eCPR between OHCA and IHCA showed no difference in outcome but higher survival rates were achieved if time to eCPR was < 75 min [15]. A current review incorporating available studies revealed a 50% survival rate in IHCA patients if eCPR initiation following cardiac arrest was < 30 min, 30% if time to eCPR was 30–60 min, and 18% if it was > 60 min. In OHCA patients, survival rates were 15–20% even if time from arrest to eCPR was < 60 min [16]. Bartos et al. demonstrated a similar correlation of neurologic intact survival and time to succesful establishment of extracorporeal circulation in a cohort of 160 OHCA patients [8]. Our results also emphasize that an overall time requirement for patient transfer, decision, and intervention, i.e.start of eCPR < 60 min was associated with higher survival rates. There are basically two strategies to improve the time interval between collaps and eCPR: 1) early decision to rapidly transfer the patient to a CAC and 2) performing eCPR pre-clinically in selected settings. While the former requires a paradigm shift for EMS teams, the latter has considerable logistical implications. A recent trial in Paris compared outcomes during two periods without (period 1) and with (period 2) implementation of a protocol that included pre-clinical start of eCPR to optimize the timing. Survival rates were higher during the second period [6]. Survival between pre- hospital and in-hospital eCPR was similar when implementation occurred within 60 min, as well as when time to implementation exceeded 60 min. Interestingly in both groups survival and CPC outcome improved from period 1 to period 2, indicating that the main effect may have been due to a more aggressive and protocol-driven approach rather than a specific mode of provision. Further analysis of the Paris data revealed somewhat conflicting results because low-flow duration greater than 60 min was not significantly associated with lower survival rates. The authors explain this by a possible selection bias in favor of in-hospital eCPR [17]. In addition, more patients in period 2 received coronary angiography and PCI.

Retrospective data from Australia revealed a high number of patients with good outcome following initiation of eCPR within 45 min of circulatory arrest in primarily IHCA patients, and pre-ECLS lactate was found to be predictive for mortality; however, the decision for eCPR was made individually without standardized inclusion criteria [18].

Current guidelines emphasize starting eCPR in selected patients within 60 min [19].

However, as our data and other results show, this timeline cannot be achieved in every patient, especially after OHCA due to many circumstances.

### Importance of neurological assessment and prognostication

Neurological assessment in our program includes repetitive neurological examination, neurophysiological testing (SSEP, EEG), circulating concentrations of neuron specific enolase (NSE; limited by hemolysis due to eCPR device) and brain imaging (CT with determination of gray-white matter ratio (GWR)). Results of all tests are taken into account for the neurological prognosis, which is not made earlier than 3 days after arrest. It should be noted, that this assesment is recommended by current guidelines only for post cardiac arrest patient with initial ROSC not undergoing eCPR. There is a strong need to define and validate the optimal neuroprognostication strategy in the subgroup of eCPR patients.

### Late ROSC

Interestingly, a relevant number of patients under ongoing CPR that were transferred to our center for eCPR were found to have regained ROSC during the admission procedure and before the start of eCPR (*n* = 31). Nine patients survived and were discharged with good neurological outcome. We consider this as a secondary benefit of implementing a 24/7 eCPR program. Although it remains speculative, we assume that in the majority of these cases, ACLS would probably have been stopped earlier at the scene if EMS would not have decided to transport the patient to our CAC for eCPR.

### Strength and limitations

Our analysis has several strength and limitations. The strength includes a comparatively large sample size of patients treated in a single center, the coverage of all patients referred for eCPR and not only those accepted, the implementation of in- and exclusion criteria, which reduces interpretation bias and the inclusion of data on outcomes after hospital discharge.

The limitations include the fact that this is a retrospective analysis of clinical routine data, the heterogeneity of the patient group with diverse causes of circulatory arrest and multiple comorbidities (mixed analyses OHCA and IHCA,Table S 2). Furthermore, data obtained pre-clinically and reported by the EMS team may be imprecise, especially with respect to time lines and time intervals and information regarding sufficient bystander CPR.

As background information regarding prior medical history as well as circumstances of the collapse frequently become uncovered after admission to the hospital unfortunately, some cases were transferred by EMS for ECMO although clearly not matching local inclusion criteria.

## Conclusion

Establishing an eCPR programm requires careful planning, skill training, substantial resources and interdisciplinary expertise. It can be life-saving for a subset of individuals with refractory circulatory arrest, with time to initiation of eCPR being a main determinant of survival.

In this investigation survivors had a shorter time from beginning of collapse to admission as well as a shorter time from beginning of collapse to the start of eCPR.

Longer timing to eCPR and lower initial pH were identified as significant independent predictors for mortality.

### Future perspectives

Highlighting the complexity of eCPR and the association of higher patient volume with improved survival, a recent position paper by The International ECMO Network (ECMONet) and ELSO recommend eCPR to be performed in high volume centers (Comprehensive Care Centers) with defined eCPR criteria and a structured post-circulatory arrest pathway [20].

Such centers are recommended to bundle expertise and improve treatment quality, resulting in a higher proportion of patients with good neurological outcome after circulatory arres [21, 22].

Along the same lines a current consensus statement of acute care societies in Germany under the patronage of the German Resuscitation Council emphasizes the importance of structure and experience and recommends a minimum case load of 30 patients undergoing eCPR per year based on an implemented pathway to ensure quality and experience [19, 20, 23, 24].

There is a strong need to define and validate the optimal neuroprognostication strategy in the subgroup of eCPR patients.

## Supplementary information

**Additional file 1: Table S1.** Detailed information concerning etiology of cardiac arrest depending of initial rhythm (survivors).

**Additional file 2: Figure S1.** Kaplane-Meiersurvival analysis, 2 patients lost of follow up+.

**Additional file 3: Table S2.** Baseline parameter according (A) location of arrest and (B) cause of arrest.

## Data Availability

All data generated or analysed during this study are included in this published article.
