# Supplementary material for: Establishment of an extracorporeal cardio-pulmonary resuscitation program in Berlin – outcomes of 254 patients with refractory circulatory arrest
Source: Scand J Trauma Resusc Emerg Med. 2020 Sep 23;28:96. doi: 10.1186/s13049-020-00787-w (PMC7513459; doi:10.1186/s13049-020-00787-w)
Supplement: Supplementary file 1 — Additional file 1: Table S1. Detailed information concerning etiology of cardiac arrest depending of initial rhythm (survivors). [file 13049_2020_787_MOESM1_ESM.docx]

Table S 1: Detailed information concerning etiology of cardiac arrest depending of initial rhythm (survivors)

| **Characteristics of EMS first rhythm (shockable vs. non-shockable) surviving patients** | | | | | |
| --- | --- | --- | --- | --- | --- |
| **variable** |  | **shockable n=11** | | **non-shockable n=7** | **p-value** |
| gender (male) |  | 9 (81.8%) | | 5 (71.4%) | n.s. |
| age (years) |  | 61 (49-66) | | 54 (31-60) | n.s. |
| OHCA |  | 5 (45.5%) | | 3 (42.9%) | n.s. |
| rhythm on admission (shockable) |  | 7 (63.6%) | | 1 (14.3%) | n.s. |
| epinephrine (mg; total amount) |  | 6 (0.75-10) | | 4 (2-6) | n.s. |
| APACHE (admission) | 39 (36-45) | |  | 39 (38-41) | n.s. |
| collapse to admission (min) | 37.5 | |  | 33 | n.s. |
| *admission laboratory values* |  | |  |  |  |
| pH | 7.25 (7.18-7.38) | |  | 7.15 (7.15-7.34) | n.s. |
| lactate | 73 (44-130) | |  | 124 (31-130) | n.s. |
| potassium | 4 (3.5-4.5) | |  | 3.9 (2.5-4.7) | n.s. |
| INR | 1.46 (1.18-1.77) | |  | 2.41 (1.28-2.6) | n.s. |
| Ventilator time | 756 (323-1060) | |  | 433 (136.25-979) | n.s. |
| cardiac cause   - myocardial infarction - rhytmogenic   non - cardiac cause  • hypothermia  • pulmonary artery embolism  • intoxication  • drown  • allergic shock | 10 (90.9%)  10  0  1 (9.1%)  1  0  0  0  0 | |  | 2 (28.6%)  1  1  5 (71.4%)  0  1  2  1  1 | < 0.05* |
| *Outcome*  CPC 1-2 | 9 (81.8%) | |  | 6 (85.7%) | n.s. |
| CPC 3-4 | 2 (18.2%) | |  | 1 (14.3%) |  |

Data are given as median (25-75% interquartile range) or absolute numbers; OHCA out-of-hospital cardiac arrest; EMS emergency medical service; APACHE Acute Physiology And Chronic Health Evaluation; INR international normalized ratio; CPC Cerebral Categories Scale
