# Supplementary figures and images for: Establishment of an extracorporeal cardio-pulmonary resuscitation program in Berlin – outcomes of 254 patients with refractory circulatory arrest
Source: Scand J Trauma Resusc Emerg Med. 2020 Sep 23;28:96. doi: 10.1186/s13049-020-00787-w (PMC7513459; doi:10.1186/s13049-020-00787-w)

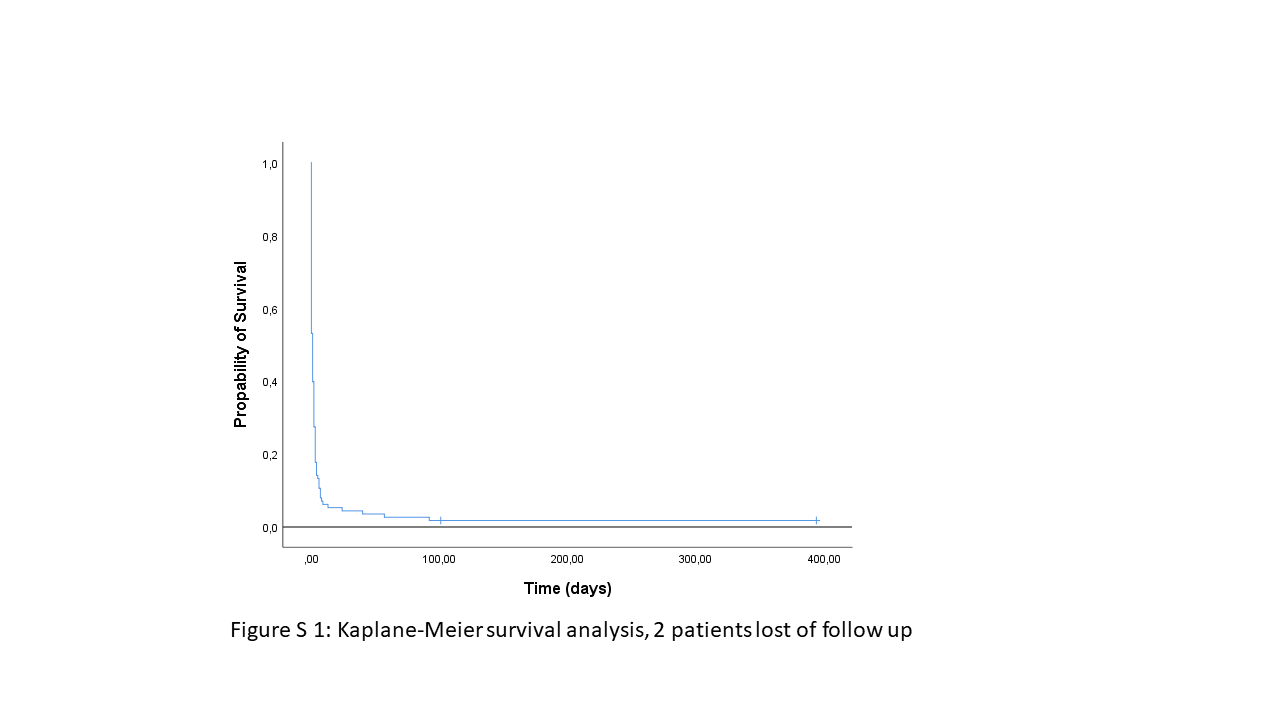

Supplement: Supplementary file 2 — Additional file 2: Figure S1. Kaplane-Meiersurvival analysis, 2 patients lost of follow up+. [file 13049_2020_787_MOESM2_ESM.tif]
