# Supplementary material for: Establishment of an extracorporeal cardio-pulmonary resuscitation program in Berlin – outcomes of 254 patients with refractory circulatory arrest
Source: Scand J Trauma Resusc Emerg Med. 2020 Sep 23;28:96. doi: 10.1186/s13049-020-00787-w (PMC7513459; doi:10.1186/s13049-020-00787-w)
Supplement: Supplementary file 3 — Additional file 3: Table S2. Baseline parameter according (A) location of arrest and (B) cause of arrest. [file 13049_2020_787_MOESM3_ESM.docx]

Table S 2: baseline parameter according (A) location of arrest and (B) cause of arrest

| 1. **Characteristics OHCA vs. IHCA** | | | | |
| --- | --- | --- | --- | --- |
| **variable** | **all**  **n= 126** | **OHCA**  **n=85** | **IHCA**  **n=41** | **p-value** |
| gender (male) | 93 (73.8%) | 71 (83.5%) | 22 (53.7%) | < 0.05 |
| age (years) | 52 (42-61) | 51 (41.5-58) | 55 (43-66) | n.s. |
| first rhythm (shockable) | 64 (51%) | 49 (57.6%) | 15 (36.6%) | n.s. |
| rhythm on admission on ICU (shockable) | 32 (25%) | 20 (23.5%) | 12 (29.3%) | n.s. |
| Epinephrine (mg; total amount) | 7 (4-10) | 7 (4,25-9) | 6 (4-14) | n.s. |
| APACHE admission | 41 (38-46) | 42 (38-46) | 41 (37-46.5) | n.s. |
| cardiac reason | 76 (60%) | 56 (65.9%) | 20 (48.8%) | n.s. |
| non-cardiac reason | 50 (40%) | 29 (34.1%) | 21 (51.2) | n.s. |
| collapse to admission (min) | 57 (45-67) | 60 (49-69) | 30 (18-48) | <0.05 |
| collapse to eCPR (min) | 88 (73-111) | 99 (80-120) | 63 (40-90) | <0.05 |
| admission laboratory values |  |  |  |  |
| pH | 7.1 (6.9-7.2) | 6.97 (6.87-7.17) | 7.15 (6.99-7.31) | < 0.05 |
| Lactate | 137 (104-173) | 141 (113-180) | 126 (74.8-161.25) | < 0.05 |
| potassium | 4.3 (3.7-5.1) | 4.3 (3.7-4.73) | 4.4 (3.8-5.25) | n.s. |
| INR | 1.8 (1.5-3.2) | 1.78 (1.53-3.24) | 1.81 (1.38-3.14) | n.s. |
| Outcome (good;%) | 15 (11.9%) | 7 (8.24%) | 8 (19.5%) | n.s. |
| Mortality (alive;%) | 18 (14.3%) | 8 (24.4%) | 10 (9.4%) | n.s. |

Data are given as median (25-75% interquartile range) or absolute numers; OHCA out-of-hospital cardiac arrest; IHCA in-hospital cardiac arrest; EMS emergency service; APACHE Acute Physiology And Chronic Health Evaluation; INR international normalized ratio; good outcome define as cerebral performance category1-2.

| 1. **Characteristics cardiac vs. non – cardiac cause** | | | | |
| --- | --- | --- | --- | --- |
| **variable** | **all**  **n= 126** | **cardiac**  **n=76** | **non – cardiac**  **n=50** | **p-value** |
| gender (male) | 93 (73.8%) | 66 (86.8%) | 27 (54%) | < 0.05 |
| age (years) | 52 (42-61) | 55 (47.3-62.8) | 47 (32.8-58) | <0.05 |
| first rhythm (shockable) | 64 (51%) | 46 (60.5%) | 18 (36%) | < 0.05 |
| rhythm on admission on ICU (shockable) | 32 (25%) | 23 (30.3%) | 9 (18%) | n.s. |
| Epinephrine (mg; total amount) | 7 (4-10) | 6.5 (4-9.3) | 7 (4-12.5) | n.s. |
| APACHE admission | 41 (38-46) | 41 (37-45) | 42 (38.5-47) | n.s. |
| collapse to admission (min) | 57 (45-67) | 60 (47-69) | 53 (41-63) | n.s. |
| collapse to eCPR (min) | 88 (73-111) | 95 (75-111) | 85 (60-111) | n.s. |
| admission laboratory values |  |  |  |  |
| pH | 7.1 (6.9-7.2) | 7.1 (6.9-7.2) | 7 (6.9-7.2) | n.s. |
| Lactate | 137 (104-173) | 138 (90.3-171) | 131 (108.5-177.5) | n.s. |
| potassium | 4.3 (3.7-5.1) | 4.3 (3.8-4.7) | 4.4 (3.7-5.7) | n.s. |
| INR | 1.8 (1.5-3.2) | 1.67 (1.38-2.46) | 2.66 (1.72-5.42) | < 0.05 |
| Outcome (good;%) | 15 (11.9%) | 10 (13.2%) | 5 (10%) | n.s. |
| Mortality (alive;%) | 18 (14.3%) | 12 (15.8%) | 6 (12%) | n.s. |

Data are given as median (25-75% interquartile range) or absolute numers; OHCA out-of-hospital cardiac arrest; IHCA in-hospital cardiac arrest; EMS emergency service; APACHE Acute Physiology And Chronic Health Evaluation; INR international normalizied ratio; good outcome define as cerebral performance category1+2.
